# Epigenetics Is Implicated in the Basis of Gender Incongruence: An Epigenome-Wide Association Analysis

**DOI:** 10.3389/fnins.2021.701017

**Published:** 2021-08-19

**Authors:** Karla Ramirez, Rosa Fernández, Sarah Collet, Meltem Kiyar, Enrique Delgado-Zayas, Esther Gómez-Gil, Tibbert Van Den Eynde, Guy T’Sjoen, Antonio Guillamon, Sven C. Mueller, Eduardo Pásaro

**Affiliations:** ^1^Laboratory of Psychobiology, Department of Psychology, Institute Advanced Scientific Research Center (CICA), University of A Coruña, A Coruña, Spain; ^2^Laboratory of Neurophysiology, Center for Biophysics and Biochemistry, Venezuelan Institute for Scientific Research (IVIC), Caracas, Venezuela; ^3^Department of Endocrinology, Ghent University, Ghent, Belgium; ^4^Department of Experimental Clinical and Health Psychology, Ghent University, Ghent, Belgium; ^5^Department of Psychiatry, Hospital Clínic, Barcelona, Spain; ^6^Department of Psychobiology, Faculty of Psychology, National University of Distance Education (UNED), Madrid, Spain

**Keywords:** DNA methylation, epigenetics, gender dysphoria, gender identity, gender incongruence

## Abstract

**Introduction:**

The main objective was to carry out a global DNA methylation analysis in a population with gender incongruence before gender-affirming hormone treatment (GAHT), in comparison to a cisgender population.

**Methods:**

A global CpG (cytosine-phosphate-guanine) methylation analysis was performed on blood from 16 transgender people before GAHT vs. 16 cisgender people using the Illumina© Infinium Human Methylation 850k BeadChip, after bisulfite conversion. Changes in the DNA methylome in cisgender vs. transgender populations were analyzed with the Partek^®^ Genomics Suite program by a 2-way ANOVA test comparing populations by group and their sex assigned at birth.

**Results:**

The principal components analysis (PCA) showed that both populations (cis and trans) differ in the degree of global CpG methylation prior to GAHT. The 2-way ANOVA test showed 71,515 CpGs that passed the criterion FDR *p* < 0.05. Subsequently, in male assigned at birth population we found 87 CpGs that passed both criteria (FDR *p* < 0.05; fold change ≥ ± 2) of which 22 were located in islands. The most significant CpGs were related to genes: *WDR45B, SLC6A20, NHLH1, PLEKHA5, UBALD1, SLC37A1, ARL6IP1, GRASP*, and *NCOA6*. Regarding the female assigned at birth populations, we found 2 CpGs that passed both criteria (FDR *p* < 0.05; fold change ≥ ± 2), but none were located in islands. One of these CpGs, related to the MPPED2 gene, is shared by both, trans men and trans women. The enrichment analysis showed that these genes are involved in functions such as negative regulation of gene expression (GO:0010629), central nervous system development (GO:0007417), brain development (GO:0007420), ribonucleotide binding (GO:0032553), and RNA binding (GO:0003723), among others.

**Strengths and Limitations:**

It is the first time that a global CpG methylation analysis has been carried out in a population with gender incongruence before GAHT. A prospective study before/during GAHT would provide a better understanding of the influence of epigenetics in this process.

**Conclusion:**

The main finding of this study is that the cis and trans populations have different global CpG methylation profiles prior to GAHT. Therefore, our results suggest that epigenetics may be involved in the etiology of gender incongruence.

## Introduction

Sexual development in mammals begins at conception, when the sex chromosome pair is determined as XX or XY. Later, the biological sex will imply differences in gonadal development, hormonal environment, sexual behavior, as well as other physical and behavioral differences. The current hypothesis about brain sexual development points to the existence of a complex “mosaic” model in the mammalian brain with a diversity of mechanisms involved, that allows a variable degree of masculinization/feminization within the brain (Joel et al., 2020).

But in humans, it is possible to differentiate between sex and gender. Whereas, gender identity could be defined as one’s innermost concept of self as male, female, a blend of both or neither (American Psychological Association, 2012; Gómez-Gil, 2019) that could be coincident or not, with the sex assigned at birth. According to this concordance between sex and gender, we can differentiate into “cisgender” or “transgender” people, respectively (Polderman et al., 2018). Gender incongruence (GI) as per International Classification of Diseases ICD-11 (World Health Organization [WHO], 2018) is characterized by a pronounced and persistent incongruence between the individual’s experience of gender and their sex assigned at birth.

According to the latest research, the origin of GI is complex and multifactorial. It might be associated with neurodevelopmental processes of the brain as well as genetic and epigenetic factors. With regards to the neuroanatomy, whereas post-mortem histological studies showed feminization of the central region of the bed nucleus of the stria terminalis in trans women (Zhou et al., 1995), more recent structural MRI studies indicated different brain phenotypes in trans women, trans men, cis women, and cis men (Guillamon et al., 2016; Kreukels and Guillamon, 2016; Nota et al., 2017; Baldinger-Melich et al., 2020; Mueller et al., 2021).

Paralleling the brain structural research, studies have been searching for a genetic component of GI. Some authors found variations in the DNA sequence of the androgen (AR) and estrogen (ERs, α and β) (Henningsson et al., 2005; Hare et al., 2009; Fernández et al., 2014a, b, 2016, 2018, 2020a; Cortés-Cortés et al., 2017; Foreman et al., 2019) that could hypothetically modulate the sensitivity of the nuclear steroid receptors. Furthermore, since AR and ER (α and β) are, at the same time, hormonal receptors and transcription factors, the modulation of gene expression via activation of AR and ERs by their ligands and coactivators, could be another presumptive mechanism underlying GI (Fernández et al., 2021; Ramírez et al., 2021).

Epigenetics offers an interesting complement to genetic studies because it reflects the interconnection between genes and the environment and could be a mechanism underlying GI given its sensitivity to environmental stimuli. Moreover, it could be possible to detect the capacity of the GAHT to modify gene expression and their stability over time.

DNA methylation (DNAm), which is the most stable and widely studied epigenetic modification to date, involves the covalent addition of a methyl group to cytosine residues adjacent to guanine in DNA (CpG sites) (Bird, 1986) and is associated with changes in gene transcription when they are located in gene promoter regions (Suzuki and Bird, 2008). Using DNA methylation analysis, epigenetic regulation has been shown to be critical in the control of sexual differentiation of the brain (McCarthy and Nugent, 2015; Nugent et al., 2015; Joel and McCarthy, 2017; Li et al., 2017; Joel et al., 2020; McCarthy, 2020). Thus, inhibition of DNA methylation in developing mice brains induces aberrant neurobehavioral profiles and disrupts sexually dimorphic neurobehavioral phenotypes in adulthood (Li et al., 2017; McCarthy, 2020). Furthermore, the sex difference in maternal anogenital licking of male compared to female pups produces a different methylation of the estrogen receptor α promoter in the preoptic area (Kurian et al., 2010).

Previous studies carried out in our laboratory in people with GI have found that certain environmental factors such as GAHT modify the methylation profile of the promoters of the ERα (Aranda et al., 2017; Fernández et al., 2020b), the AR and the ERβ (Aranda et al., 2017). Aranda et al. (2017) found no differences in the DNA methylation of the ERα in trans women, while DNA methylation was increased in trans men at 6 and 12 months of GAHT. The AR showed a significant increase of methylation in trans women after 12 months of estrogen supplementation. With respect to the ER α promoter, before the hormone treatment, trans men showed a lower methylation level with respect to both cis men and women, whereas trans women reached an intermediate methylation level with respect to the cis groups. However, after 6 months of GAHT, trans men showed a methylation increase, and both transgender groups reached a midway methylation level between cis men and cis women (Fernández et al., 2020b). Thus, both studies suggest that epigenetic changes in the sex steroid receptor promoters might be associated to GAHT. In fact, 6 months of GAHT was sufficient to modulate epigenetic changes at the estrogen and androgen receptor promoter regions. Yet, these prior studies focused exclusively on the AR and ER receptors and, to our knowledge, a global CpG analysis has not been performed to date in people with GI.

Therefore, taking into account our previous analyses and to achieve a broader perspective of the influence of epigenetics in GI, our main objective of this study was to carry out a global CpG methylation analysis in a transgender population before GAHT and cisgender comparisons, assessed by ethnicity, geographical origin and sex.

## Subjects and Methods

### Study Participants and Experimental Design

We analyzed sixteen Flemish Belgian transgender people (9 trans men and 7 trans women) before GAHT, and sixteen Flemish Belgian cisgender people (8 cis men and 8 cis women). The population was recruited at the Center for Sexology and Gender, Dept. of Endocrinology at the Ghent University Hospital (Belgium).

To obtain a homogeneous population avoiding stratification (Michels et al., 2013), the following inclusion and exclusion criteria were applied: for transgender people were the presence of GI according to ICD-11 (World Health Organization [WHO], 2018), identification with the other sex (male or female); and no prior history of hormonal treatment.

The exclusion criteria were the presence of psychiatric, neurological and hormonal diseases, and major medical condition. To the cisgender population the same exclusion criteria were applied. The mean age of the cisgender group at the beginning of the investigation study was 27.75 (SD ± 7.6)years and 34.1 (SD ± 14.0) years for the transgender group. Written informed consent was obtained from all participants after a full explanation of the procedures. The study was approved by the Ethical Committees of Gent University Hospital and UNED.

### Genomic DNA Methylation Analysis

Genomic DNAs were extracted from peripheral blood samples using the DNeasy Blood and Tissue Kit from Qiagen following the manufacturer’s protocol, and an aliquot of 1 μg DNA per subject was processed for bisulfite conversion (Zymo Research EZ Methylation Kit), according to the manufacturer’s instructions.

DNA methylome was analyzed using the Illumina© Infinium Human Methylation 850k BeadChip array (Illumina, San Diego, CA, United States) that assesses 862,927 cytosine–phosphate–guanine (CpG) sites throughout the genome, covering 99% of RefSeq genes, 95% of CpG islands and high coverage of enhancer regions. In this study we selected the CpGs located in islands because they often coincide with promoters (Illingworth and Bird, 2009), and methylation modification of the promoter regions has the capacity to modify gene expression (Maurano et al., 2015) because methylation of the promoters prevents the binding of RNA polymerases and/or other diverse transcriptional factors to the promoter region, thereby inhibiting DNA transcription (Kang et al., 2019).

Beadchips were scanned with the Illumina iScan SQ system, and image intensities were extracted with the Genome Studio (2011.1). DNA quality checks, data normalization, and statistical filters were performed with the Partek^®^ Genomics Suite^®^ v7.19.1018 Methylation Module. Probes from the X and Y chromosomes were excluded from the study, and probes based on detection *P* > 0.05 were also filtered to exclude low-quality probes. Analysis of differentially methylated loci in humans and mice often excludes probes on the X and Y chromosomes because of the difficulties caused by the inactivation of one X chromosome in female samples.

**FIGURE 1:**
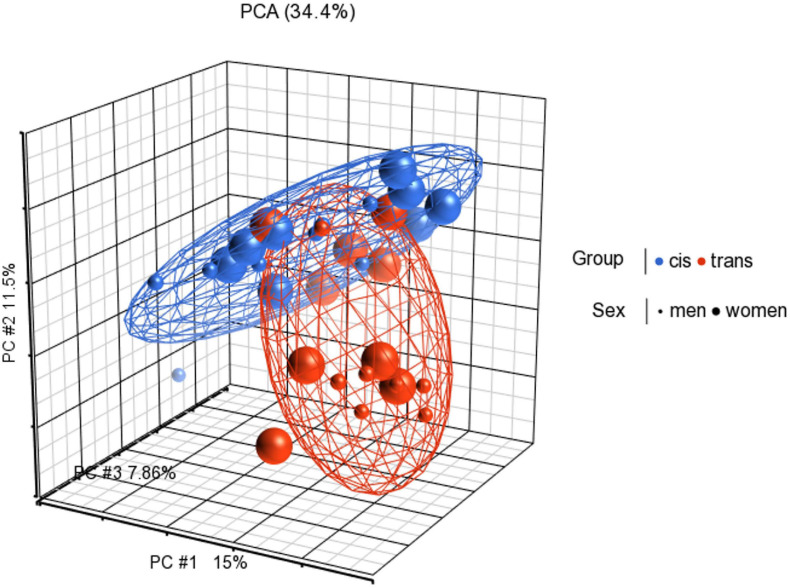
Principal components analysis (PCA) in 3D showing methylation profiles of the study samples. Each sample is represented by a dot, the axes are the first three PCs, the percentages indicate the fraction of variance explained by each PC. The number at the top is the variance explained by the first three PCs. The samples are colored according to the levels of the variable “group” (blue for cisgender individuals and red for transgender individuals), and sized according to the levels of the variable “sex assigned at birth” (small for men and big for women).

Functional normalization, NOOB background correction and dye correction were applied. Principal component analysis (PCA) was performed to visualize clusters in the methylation data, and as a quality control procedure (Figure 1).

The raw methylation score for each probe was represented as methylation beta (β), in which β = intensity of the methylated allele (M)/intensity of the unmethylated allele (U) + intensity of the methylated allele (M) + 100. β-values range from 0 (unmethylated) to 1 (fully methylated) and can be broadly interpreted as the percentage of CpG methylation (Bibikova et al., 2011; Moran et al., 2016). Subsequently β-values were converted to *M*-values using the following equation: *M*-value = log2(β/(1 −β)). An *M*-value close to 0 for a CpG site indicates a similar intensity of the methylated and unmethylated probes, which means the CpG site is about half-methylated. Positive *M*-values mean that more molecules are methylated than unmethylated, while negative *M*-values mean that more molecules are unmethylated than methylated. As discussed by Du et al. (2010), the β-value has a more intuitive biological interpretation, but the *M*-value is more statistically valid for the differential analysis of methylation levels. Because we were performing differential methylation analysis, Partek Genomics Suite automatically created the *M*-values to use for statistical analysis. Distribution of *M*-values across the samples was inspected by a box-and-whiskers plot and the distribution of beta-values by a histogram.

Differential methylation analyses (mean M variation, ΔM) aimed to evaluate methylation differences between the studied groups. Individual probes were then filtered based on Illumina detection *P* < 0.05 value, and a false discovery rate correction (FDR) *p* < 0.05 and a fold change ≥ ± 2 were applied.

All analyses were done by the Partek^®^ Genomics Suite^®^ software, version 7.0. The human reference genome (GRCh37/hg19 assembly) was used to determine the location and features of the gene region using the UCSC Genome Browser (Kent et al., 2002).

### Statistical Analysis

**FIGURE 2:**
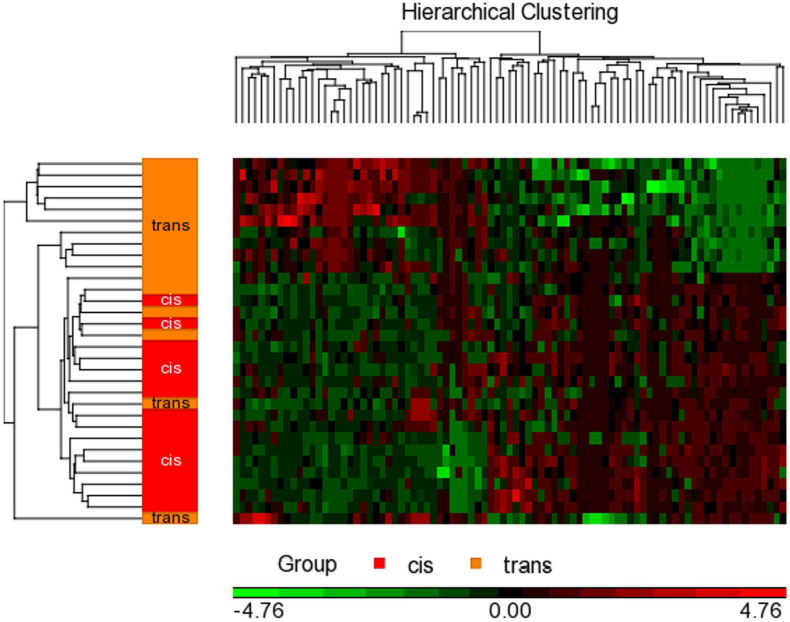
Hierarchical clustering with heat map invoked on the list of significant CpGs FDR *p* < 0.05 and a fold change ≥ 2.0 in male assigned at birth populations. The experimental groups are rows, while the CpGs from the cisgender vs. transgender spreadsheet are columns. CpGs with higher methylation are colored red, CpGs with lower methylation are colored green. Samples of transgender people are colored orange and samples of cisgender people are colored red in the dendrogram on the left-hand side of the heat map.

To detect the differential methylation in global CpGs that varies across all samples we performed a 2-way ANOVA test comparing cisgender vs. transgender individuals by their sex assigned at birth. Then, we added two contrast interaction terms to find those genes that specifically change in each group: we contrasted cis men vs. trans people with male sex assigned at birth (trans women), and cis women vs. trans people with female sex assigned at birth (trans men). For each contrast, a *P*-value, Beta difference (Δβ), and M difference (ΔM) were generated. Hierarchical cluster analysis of the significant CpGs was carried out with the Heatmap function in the Partek^®^ Genomics Suite^®^ 7.0 (Figure 2). *P*-values were calculated using false discovery rate correction for multiple comparisons, FDR *p* < 0.05; corrected, two-tailed, and fold change ≥ ± 2).

### Controls Applied to Exclude Genes Associated With Smoking and Age

The most robustly validated findings to date with DNA methylation studies have been the association between DNA methylation in blood and smoking. The genes that have shown the strongest associations to smoking status are: *AHRR*, 2q37.1, 6p21.33, *F2RL3, GPR15, GFI1, CYP1A1, MYO1G*, and *CNTNAP2* (Flanagan, 2015). We have used this knowledge to create a list of genes related to smoking status that was checked from our list of genes related to our variable of interest. This has been done because methylation alterations are detectable in blood DNA even in ex-smokers who stopped smoking up to 10–20 years before (Flanagan, 2015).

Furthermore, several genes appear consistently associated with age, including *ELOVL2, CCDC102B, OTUD7A*, and *FHL2*. Therefore, we have used this list of genes to exclude them from our study, prior to the enrichment analysis (Flanagan, 2015).

### Functional and Regulatory Enrichment Analysis

The distribution of significant CpGs differentially methylated in females and males was examined across functional and regulatory annotations. CpG findings were mapped to known genes for enrichment of Gene Ontology (GO) classifications. The GO analysis and pathway enrichment analysis were carried with the Partek^®^ Pathway program and the WebGestalt (WEB-based Gene SeT AnaLysis Toolkit)^1^ (Liao et al., 2019) using the Genomes (KEGG) and the Panther databases. The GO ontology includes three independent divisions: biological process (BP), molecular function (MF) and cellular component (CC). The biological process can be defined as a biological objective to which the gene or gene product contributes. The molecular function is defined as the biochemical activity of a gene product, while the cellular component refers to the place in the cell where the gene product is active (Draghici, 2012).

## Results

### Analysis of 2-Way ANOVA Test

When we compared the DNAm of transgender and cisgender populations by the variable sex assigned at birth, we found a baseline of 71,515 CpGs that passed the criterion FDR *p* < 0.05. Furthermore, 28.5% were in islands. About a third of these positions (32.3%) were hypomethylated while 67.66% were hypermethylated in cis men. In cis women, 27.05% of the CpGs were hypomethylated, while 72.95% were hypermethylated. These statistically significant CpGs were distributed among all autosomes.

### Analysis of Cis Men vs. Trans Women

**FIGURE 3:**
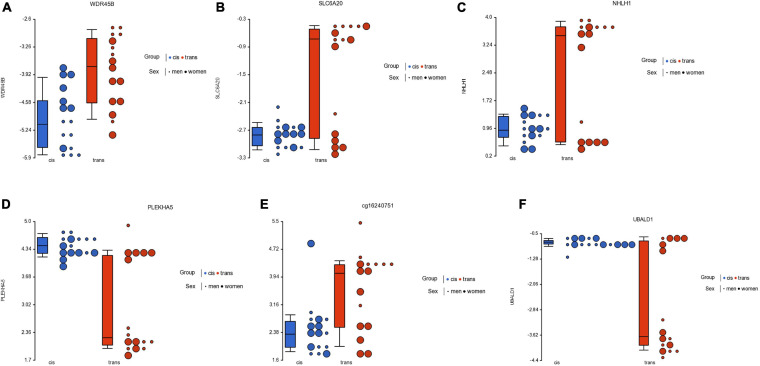
Dot plot showing *M*-value data for genes *WDR45B* **(A)**, *SLC6A20* **(B)**, *NHLH1* **(C)**, *PLEKHA5* **(D)**, one intergenic locus (cg16240751) **(E)**, and *UBALD1* **(F)**, for cisgender vs. transgender populations by their sex assigned at birth. Each sample is represented by a dot, which corresponds to the overall degree of methylation (*M*-value data). The samples are colored according to the levels of the variable “group” (blue for cisgender population and red for transgender population), and sized according to the levels of the variable “sex assigned at birth” (big for women and small for men). The middle line is the median, the box represents the upper and the lower quartile, while the whiskers correspond to the 90th and 10th percentiles of the data.

Subsequently, when we specifically contrasted the methylome in people who were male assigned at birth, we found 87 CpGs that passed both criteria (FDR *p* < 0.05; fold change ≥ ± 2), of which 22 CpGs were located in islands: 14 were hypomethylated while 8 were hypermethylated in the cis population. Table 1 lists the 22 CpG islands in populations assigned male at birth that passed both criteria. The most significant CpG islands were related to genes: *WDR45B, SLC6A20, UBALD1, GRASP, NHLH1, PLEKHA5, SLC37A1, NCOA6*, and *ARL6IP1* (Figure 3).

**TABLE 1:** The 22 CpG islands that passed statistical correction (FDR *p* < 0.05; fold change ≥ 2.0), in the population assigned male at birth.

**Probeset ID**	**Gene symbol**	***P*-value (cis vs. trans)**	**Difference (cis vs. trans)**	**Difference (description) (cis vs. trans)**
cg10401531	*WDR45B*	2.97E-07	−2.20167	Cis men down vs. trans women
cg09700085	*SLC6A20*	3.49E-05	−2.00023	Cis men down vs. trans women
cg21538190	*NHLH1*	4.81E-05	−2.47591	Cis men down vs. trans women
cg24441383	*PLEKHA5*	4.87E-05	2.03159	Cis men up vs. trans women
cg16240751	*—*	5.29E-05	−2.05596	Cis men down vs. trans women
cg25764197	*UBALD1*	6.39E-05	2.6023	Cis men up vs. trans women
cg12993026	*SLC37A1*	0.000286382	2.19509	Cis men up vs. trans women
cg26358144	*ARL6IP1*	0.000298896	−2.10562	Cis men down vs. trans women
cg09016212	*GRASP*	0.000450551	2.40079	Cis men up vs. trans women
cg04208499	*NCOA6*	0.00138631	−5.96574	Cis men down vs. trans women
cg11502198	*ABT1*	0.00144373	−2.00222	Cis men down vs. trans women
cg02090742	*C17orf79*	0.00158261	−5.81018	Cis men down vs. trans women
cg11738485	*HOOK2*	0.00166823	−4.62858	Cis men down vs. trans women
cg04657146	*HOOK2*	0.00199853	−3.6818	Cis men down vs. trans women
cg09698465	*—*	0.00229726	6.38536	Cis men up vs. trans women
cg14623093	*GORASP1*	0.00285936	2.37051	Cis men up vs. trans women
cg20544675	*LETM2*	0.00336149	2.15344	Cis men up vs. trans women
cg12688781	*AACS*	0.00360693	−2.22118	Cis men down vs. trans women
cg01655658	*HLA-L*	0.00371637	−2.56482	Cis men down vs. trans women
cg11424828	*MYOM2*	0.00407032	−4.38544	Cis men down vs. trans women
cg05528899	*—*	0.00437277	−3.36645	Cis men down vs. trans women
cg24418853	*PTPLA*	0.00463796	3.29113	Cis men up vs. trans women

*(—) Some CpGs are not located in genes, which means these signature probes may be located in intragenic areas or information about them remains unknown.*

### Analysis of Cis Women vs. Trans Men

With respect to the population with female sex assigned at birth, we found 70 CpGs that passed the criterion FDR *p* < 0.05, of which 2 CpGs also passed the criterion fold change ≥ ± 2 (Table 2), but none were in islands. Table 2 lists the 2 CpGs that passed both criteria. The two significant CpGs were cg23944405 related to gene *MPPED2*, and cg16149820 that may be in intragenic areas or information about it remains unknown.

**TABLE 2:** The 2 CpGs that passed statistical correction (FDR *p* < 0.05; fold change ≥ 2.0) in the population assigned female at birth.

**Probeset ID**	**Gene symbol**	**Relation_to_UCSC_ CpG_Island**	***P*-value (cis vs. trans)**	**Difference (cis vs. trans)**	**Difference (description)** **(cis vs. trans)**
cg16149820	—	N_Shelf	3.14E-07	5.38516	Cis women up vs. trans men
cg23944405	MPPED2	N_Shelf	2.85E-06	−5.52554	Cis women down vs. trans men

*The cg16149820 CpG is not located in a gene (—), which means this signature probe may be located in intragenic areas or information about it remains unknown. N_Shelf: Location relative to the CpG island, between 2 and 4 kb up- and downstream.*

### Functional and Regulatory Enrichment Analysis

Once the significant CpGs had been selected, and prior to making the enrichment analysis, we excluded the list of genes involved in age and smoking. Subsequently, the enrichment analysis was done with the Partek^®^ Gateway program and the WebGestalt. The results of the enrichment tests yielded significant over-representation for the categories of biological process, cellular component, and molecular function ontologies. Among the main molecular functions, we can highlight: negative regulation of gene expression (GO:0010629), central nervous system development (GO:0007417), brain development (GO:0007420), purine nucleotide binding (GO:0017076), ribonucleotide binding (GO:0032553), RNA binding (GO:0003723), and ATP binding (GO:0005524), among others (Table 3).

**TABLE 3:** The results of the enrichment analysis for the categories of biological process, cellular component, and molecular function ontologies.

**Gene set**	**Description**	**Size**	**Expect**	**Ratio**	***P*-value**	**FDR**
**Enrichment categories: geneontology_biological_process**						
GO:1901566	Organonitrogen compound biosynthetic process	1,776	752.54	1.2544	0	0
GO:0010629	Negative regulation of gene expression	1,733	734.32	1.3318	0	0
GO:0009719	Response to endogenous stimulus	1,595	675.85	1.2384	0	0
GO:0007417	Central nervous system development	949	402.12	1.3727	0	0
GO:0009894	Regulation of catabolic process	875	370.76	1.3944	0	0
GO:0043604	Amide biosynthetic process	766	324.58	1.3957	0	0
GO:0031329	Regulation of cellular catabolic process	764	323.73	1.3870	0	0
GO:0007420	Brain development	714	302.54	1.3816	0	0
GO:0043043	Peptide biosynthetic process	636	269.49	1.4138	0	0
GO:0006412	Translation	613	259.75	1.4206	0	0
**Enrichment categories: geneontology_cellular_component**						
GO:0005783	Endoplasmic reticulum	1,861	620.62	1.397	0	0
GO:0031984	Organelle subcompartment	1,661	553.92	1.4768	0	0
GO:0044433	Cytoplasmic vesicle part	1,462	487.55	1.5014	0	0
GO:0042175	Nuclear outer membrane-endoplasmic reticulum membrane network	1,072	357.5	1.3958	0	0
GO:0005789	Endoplasmic reticulum membrane	1,049	349.83	1.3978	0	0
GO:0099503	Secretory vesicle	976	325.48	1.4133	0	0
GO:0005773	Vacuole	760	253.45	1.5348	0	0
GO:0000323	Lytic vacuole	670	223.43	1.5217	0	0
GO:0005764	Lysosome	669	223.1	1.524	0	0
GO:0044437	Vacuolar part	552	184.08	1.5373	0	0
**Enrichment categories: geneontology_molecular_function**						
GO:0017076	Purine nucleotide binding	1,865	750.79	1.4039	0	0
GO:0032553	Ribonucleotide binding	1,865	750.79	1.3959	0	0
GO:0032555	Purine ribonucleotide binding	1,850	744.75	1.4005	0	0
GO:0035639	Purine ribonucleoside triphosphate binding	1,786	718.99	1.4048	0	0
GO:0008144	Drug binding	1,707	687.18	1.3417	0	0
GO:0042802	Identical protein binding	1,696	682.75	1.3050	0	0
GO:0003723	RNA binding	1,603	645.32	1.3962	0	0
GO:0030554	Adenyl nucleotide binding	1,522	612.71	1.4183	0	0
GO:0032559	Adenyl ribonucleotide binding	1,509	607.47	1.4141	0	0
GO:0005524	ATP binding	1,453	584.93	1.4156	0	0

## Discussion

The main finding of this study is that the cis and trans populations have different global CpG methylation profiles, prior to GAHT. The PCA analysis showed that the spatial representation of the global methylation of these populations clearly differs between them. When comparing male sex assigned at birth individuals (cis men vs. trans women), 22 CpGs with significant methylation were located in islands. However, with respect to female assigned at birth individuals, significant changes of methylation in only 2 CpGs were found, and none were in islands. Furthermore, one of these CpGs, related to the *MPPED2* gene, is shared by both, trans men and trans women. Among the most statistically significant CpGs, we found that at least four of these genes were clearly involved in brain development and neurogenesis. These genes are *SLC6A20, PLEKHA5, NHLH1*, and *MPPED2*. Overall, our results suggest that these genes could be involved in brain development, and that epigenetic factors play a role in a differential development that might be related to GI.

When comparing cis men vs. trans women 87 CpGs passed statistical correction (FDR *p* < 0.05; fold change ≥ ± 2), of which 22 CpGs were located in islands: 14 were hypomethylated and 8 were hypermethylated in the cis population. In this study we have considered CpGs islands because they often coincide with promoter areas, and they have the capacity to modify gene expression (Maurano et al., 2015).

The most significant CpGs in trans women were related to genes *WDR45B, SLC6A20, NHLH1, PLEKHA5, UBALD1, SLC37A1, ARL6IP1, GRASP, NCOA6, ABT1*, and *C17orf79* (Table 1 and Figure 3). Among the most statistically significant CpGs, at least four of these genes were involved in brain development and neurogenesis (*WDR45B, SLC6A20, NHLH1*, and *PLEKHA5*) and three were related to transcriptional functions (*NHLH1, NCOA6*, and *ABT1*). Furthermore, the gene *C17orf79* is related to chromatin organization and its activation stimulates the transcription of the AR. Finally, another two genes were related to glutamate synapses (*ARL6IP1* and *GRASP*).

When we analyzed specifically the functions of each gene, we found that *WDR45B* is a component of the autophagy machinery that controls the major intracellular degradation process by which cytoplasmic materials are packaged into autophagosomes and delivered to lysosomes for degradation. Experiments with knockout (KO) mice exhibit many swollen axons and show cerebellar atrophy (Ji et al., 2020). On the other hand, the gene *SLC6A20* synthetizes an amino acid transporter as proline and is a regulator of brain glycine levels. Recent studies have reported that this gene is highly expressed in various brain regions and is also highly expressed in astrocytes and microglia, but only modestly expressed in glutamate or minimally in GABAergic neurons (Bae et al., 2021). This could suggest that *SLC6A20* proteins act as the regulator of both proline and glycine homeostasis in the brain.

The gene *NHLH1* is involved in neurogenesis that encodes a helix-loop-helix (HLH) protein that belongs to a family of transcription factors, some of which have been shown to play an important role in the growth and development of a wide variety of tissues. This protein is mainly expressed in the brain, specifically in the cerebellum. Ware et al. (2016) proposed that *NHLH1* is a neuronal marker. Its function might be regulating the expression of specific neuronal genes at the level of the first neurons, establishing the early axon scaffold tracts.

On the other hand, *PLEKHA5* is related to cell migration and cell to cell interactions and might also be a mediator of the brain homing phenotype (Eisele et al., 2015). Yamada et al. (2012) demonstrated that this gene may play an important role in mouse brain development. We also found differences in the methylation profile of the *UBALD1* gene, but its function is still unknown, however, it was associated with IL-8 secretion and NF-kappa-B signaling (Frenkel et al., 2019).

With respect to gene *NCOA6*, the protein encoded by this gene is a transcriptional coactivator that can interact with nuclear hormone receptors to enhance their transcriptional activator functions. It is a nuclear receptor coactivator that directly binds nuclear receptors such as for steroids (glucocorticoid receptors GR and ERs) and stimulates transcriptional activities in a hormone-dependent fashion (Eyster, 2016). Gene ontology annotations related to this gene include chromatin binding and transcription coactivator activity. Besides that, previous DNA analysis of polymorphisms related to SRC-1 and SRC-2 coactivators have pointed out their possible implication in the process of brain dimorphism (Fernández et al., 2021).

A further point in relation to this subject is that studies in mice suggest that the protein encoded by the gene *ABT1* is likely to activate basal transcription from class II promoters by interaction with the class II promoter DNA. GO annotations related to this gene include transcription coactivator activity, DNA binding, RNA binding, transcription coactivator activity, or regulation of transcription by RNA polymerase II among others.

On the other hand, when we compared cis women vs. trans men, we found significant methylation in only 2 CpGs, and none were in islands. The Venn analysis showed that one of the significant CpGs was shared by both trans groups. Thus, the cg23944405, located in the *MPPED2* gene (Metallophosphoesterase Domain Containing 2) showed statistically significant changes in methylation in trans men and trans women. This gene is expressed in most human tissues, also in the brain, both in cis men and cis women, and is expressed predominantly in fetal brains. Furthermore, Liguori et al. (2012) characterized *MPPED2* expression in human tissues of neuronal origin, and demonstrated that *MPPED2* expression is modulated during development, attributing to this gene an important role in the processes of neuronal differentiation that occur at the embryonic stage during CNS development. This gene has also been associated with altered inflammation and adverse clinical outcomes in severe blunt trauma (Schimunek et al., 2019). Furthermore, the functional importance of *MPPED2* regulation is related to cell cycle inhibition as it induces apoptosis and differentiation of neuronal precursors (Liguori et al., 2012).

**FIGURE 4:**
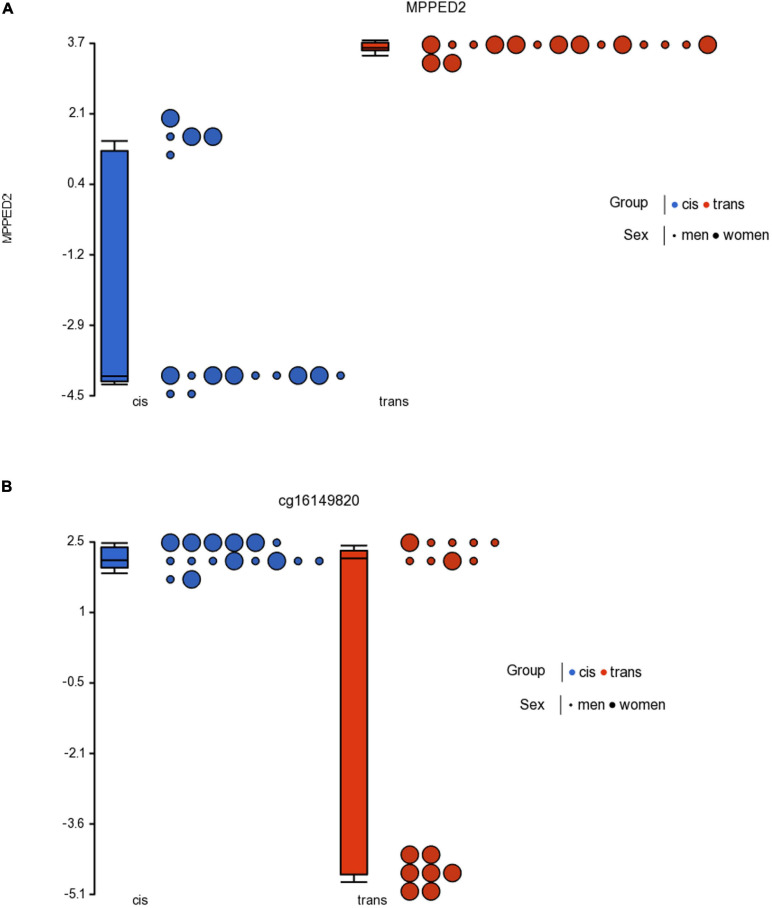
Dot plot showing *M*-value data for cg23944405 **(A)** in the *MPPED2* gene and cg16149820 **(B)** for cisgender vs. transgender populations by sex assigned at birth. Each sample is represented by a dot. The samples are colored according to the levels of the variable “group” (blue for cisgender population and red for transgender population), and sized according to the levels of the variable “sex assigned at birth” (big for women and small for men). The middle line is the median, the box represents the upper and the lower quartile, while the whiskers correspond to the 90th and 10th percentiles of the data.

Cg23944405 related to the *MPPED2* gene is hypermethylated in both trans populations (Figure 4). But this CpG is not located in an island (Table 2), so we cannot conclude that the hypermethylation in the transgender population was related to a low gene expression. Nevertheless, overall, we must point out that low metallophosphoesterase activity (*in vitro*) may play a role in the development of the CNS.

Our previous studies on the genetic basis of GI pointed to the existence of DNA sequences that modulate the sensitivity of the estrogen and androgen receptors in the trans population. Furthermore, we must remember that these nuclear receptors (ER and AR) are at the same time transcription factors, that modulate gene expression. Furthermore, the direct induction of gene expression through the activation of estrogen receptors and the androgen receptor is the presumed route for masculinization of the brain (Sato et al., 2004; Kudwa et al., 2006).

Now, with the present investigation, another small step is taken to increase our knowledge about GI. The results obtained here tell us that also epigenetics also plays an important role in the etiology of GI. Specifically, the differential methylation of essential genes in brain neurodevelopment such as *SLC6A20, PLEKHA5, NHLH1*, and *MPPED2*, are also involved in the etiology of GI. These are genes that play an important role in brain neurodevelopment, gene expression, and neuronal migration, which makes it possible to consider the existence of characteristic methylation profiles in the trans population.

In summary, our data reaffirm the hypothesis of a complex origin of GI, as the result of a combination of multiple factors such as hormones, hormone receptors, genetics and now also epigenetics.

## Limitations

A potential limitation is that the methylation data was generated for only 32 participants. To make our study more robust, it would be advantageous to repeat this in a larger sample size or validate the conclusions with a new analysis from another trans population with similar characteristics. Also to make it even stronger it would be advantageous to do a longitudinal study that we are also collecting.

Another limitation of our study is that other factors with a known influence on the DNA methylome exist that must be taken into account. For example: sleep profile (Lahtinen et al., 2019), active/sedentary lifestyle (Voisin et al., 2015), nutritional habits (Kadayifci et al., 2018), or life adversity (Cecil et al., 2020).

The effect of sleep deprivation on transcriptome and methylome has previously been studied both in experimental animal models (Lahtinen et al., 2019). Sleep deprivation induces notable changes in the brain transcriptome of rats, affecting protein synthesis, synaptic plasticity, and metabolism (Cirelli and Tononi, 2000; Cirelli et al., 2004).

Moreover, nutrition is another important factor which plays a direct role in DNA methylation (Kadayifci et al., 2018). It is believed that nutrition affects the epigenetic regulation of DNA methylation by altering the substrates and cofactors that are necessary for DNA methylation, and also by changing the activity of enzymes that regulate the one-carbon cycle, and has a role in DNA demethylation activity too.

On the other hand, multiple studies in animals and also in humans have supported a link between early adversity and DNA methylation. The first piece of evidence for the impact of early adversity on the epigenome stemmed from research in animals. In a series of seminal studies based on rodents, Weaver et al. (2004, 2005) found that variations in maternal care during the first week of life led to long-term changes in the pup’s epigenetic regulation of the glucocorticoid receptor gene (Nr3c1), a gene crucially implicated in HPA axis function. These epigenetic changes stably altered Nr3c1 expression, resulting in variations in the density of glucocorticoid receptors in the brain as well as inter-individual differences in the pup’s physiological and behavioral responses to future stressors (Turecki and Meaney, 2016; Cecil et al., 2020).

Since these and other factors have not been taken into account in our study, together with the small sample size, we believe that our study constitutes a preliminary analysis of the influence of epigenetics on gender incongruence.

## Conclusion

In conclusion, we have identified two global CpG methylation profiles in cis and trans populations, prior to gender affirming hormonal therapy. These epigenetic changes in DNAm were associated with several genes related to crucial processes during development. Moreover, these methylation data, along with our previous genetic data, support the hypothesis that GI is a complex multifactorial trait, involving intricate interactions between sex steroids, sex steroid receptors, genetics and epigenetics. This supports the view that combining genetic and epigenetic approaches in parallel may be a successful approach to understanding the mechanisms underlying brain dimorphism. Furthermore, this hypothesis is consistent with the current complex “mosaic” model of the masculinization/feminization of the brain (Joel, 2021).

## Data Availability Statement

The datasets presented in this study can be found in online repositories. The names of the repository and accession number(s) can be found below: https://www.ncbi.nlm.nih.gov/, GSE173382.

## Ethics Statement

The studies involving human participants were reviewed and approved by the Ethical Committees of Gent University Hospital and UNED. The participants provided their written informed consent to participate in this study.

## Author Contributions

AG, EP, RF, and SM contributed to the conception and design of the study. SC and MK recruited the population, involved in the study, and collected data from participants. SC and TV performed DNA extractions. RF and KR performed the statistical analysis and wrote the first draft of the manuscript. All authors contributed to manuscript revision, read, and approved the submitted version.

## Conflict of Interest

The authors declare that the research was conducted in the absence of any commercial or financial relationships that could be construed as a potential conflict of interest.

## Publisher’s Note

All claims expressed in this article are solely those of the authors and do not necessarily represent those of their affiliated organizations, or those of the publisher, the editors and the reviewers. Any product that may be evaluated in this article, or claim that may be made by its manufacturer, is not guaranteed or endorsed by the publisher.

## Footnotes

1
http://www.webgestalt.org
